# Analysis of Non-Destructive Indicating Properties for Predicting Compressive Strengths of *Dendrocalamus sericeus* Munro Bamboo Culms

**DOI:** 10.3390/ma16041352

**Published:** 2023-02-05

**Authors:** Panumat Tangphadungrat, Chayanon Hansapinyo, Chinnapat Buachart, Teewara Suwan, Suchart Limkatanyu

**Affiliations:** 1 Department of Civil Engineering, Faculty of Engineering, Chiang Mai University, Chiang Mai 50200, Thailand; 2 Excellence Center in Infrastructure Technology and Transportation Engineering, Department of Civil Engineering, Faculty of Engineering, Chiang Mai University, Chiang Mai 50200, Thailand; 3 Department of Civil and Environmental Engineering, Prince of Songkla University, Songkhla 90110, Thailand

**Keywords:** compressive load capacity, compressive strength, linear regression, bamboo

## Abstract

Bamboo is a natural material with the potential for being used in sustainable construction. However, uncertainty in the bearing capacity of the bamboo results in nonstandard values for structural engineering design. This research studied the simple and multiple linear regression analyses for indicating properties to predict the axial compressive load capacity and strength of bamboo culms, which will be useful information for quality control during building construction and further structural grading. First, twelve basic physical properties were measured from 111 samples of *Dendrocalamus sericeus* Munro bamboo culms, and axial compressive load tests of the bamboo culms were performed. Then, the correlation significance of the physical properties to axial load capacity and strength (load per area) were analyzed by the Pearson correlation method. The results show that five parameters, i.e., linear mass, culm wall thickness, external diameter, moisture content, and density, were statistically significant with the responses (compressive load capacity and compressive strength) and then defined as “candidates for indicating properties”. Next, simple linear and multiple linear regression were analyzed to formulate the relationship between the significant indicating properties and the responses. From the simple linear regression analysis, linear mass can be best used as an indicating property for predicting the maximum compressive load. The maximum compressive strength was well associated with density. The multiple linear regression analysis shows an improvement in the response predictions with respect to the simple linear regression analysis with the higher R^2^-values. Finally, structural grading of the bamboo is determined.

## 1. Introduction

Bamboo is one of the monocotyledon grass families with the tallest stems found in tropical regions. Currently, there are 1662 species of bamboo, divided into 121 genera [1]. Bamboo has a vital economic role in many developing countries, particularly in Asia. In Thailand, a country in Southeast Asia, there are a total of 15 genera and 80 species. Normally, bamboo is used to manufacture furniture as well as scaffolding [2,3]. Bamboo has been used as a construction material for centuries due to its mechanical properties suitable for structural applications, e.g., high strength-to-weight ratio, flexibility of the fibrous microstructure, low cost, faster production, and simple manufacturing processes [4,5,6,7]. One of the benefits of using bamboo as a construction material is its sustainability. Bamboo grows quickly and can be harvested and replanted with minimal impact on the environment. It is also a renewable resource, meaning it can be replenished through natural processes. Bamboo offers environmental benefits over other conventional construction materials, e.g., steel and concrete, with lower Global Warming Potential (GWP) values [8,9]. With the merit of high flexibility, using bamboo for structural elements reduces building damage under an earthquake attack [10,11,12,13] and other extreme loads [14,15,16,17].

Hailemariam et al. [18] identified barriers, benefits, and opportunities of using bamboo materials for structural purposes. A significant barrier obstructing the use of bamboo for structural elements is the difficulty in standardizing it, as with steel or concrete. As a natural material, its engineering properties, shapes, sizes, etc., are uncertain. Bamboo is not widely available in all parts of the world, and it may be difficult to obtain a consistent supply of high-quality bamboo for construction projects. When considering these factors for strength-affecting factors, experimental testing for load-bearing capacity during the design stage and quality construction control are critical. In the material testing standard, ISO 22157-1: 2019 [19] defines the test procedure for the following properties: moisture content, density (mass by volume), shrinkage, compression, bending, shear, and tension. Depending on the level of quality assurance and property invariance, the number of samples to be tested can be large. Hence, the quality control process with the tests delays the construction in practice. ISO 19624: 2018 [20] provides a method for estimating the mechanical properties of bamboo using indicating properties coupled with statistical analysis, which are much easier to measure, making it possible to reduce the test process and time.

The indicating properties are properties or measurements that can be measured non-destructively [20,21], and hence the properties can be easily obtained. The general indicative properties of bamboo are moisture content, node and internode inclusion, density, culm wall thickness, diameter, and linear mass. The lower the moisture content in bamboo culms, the higher their density and strength [22,23]. Along its height, the moisture content of bamboo decreases from the bottom to the top position, resulting in the highest strength at the top position. The moisture content usually ranges between 10% and 15% and must be kept in this range to optimize the mechanical properties [24]. The node and internode are unique characteristics of bamboo. The node decreases the tensile strength, but it does not affect other loading capacities [25,26,27]. The fiber density made up of sclerenchyma tissues affects the strength of bamboo. The higher the height position considered, the more tightly compressed the sclerenchyma tissues will be, resulting in higher strength at the top. Density, culm wall thickness, and diameter are directly related to each other, with larger sizes or volumes affecting strength [21,28,29,30]. Finally, linear mass is one of the parameters, which is the mass divided by the length. The larger the value of the linear mass, the more strength can be obtained [21,29,31,32,33]. The linear mass has the strongest correlation with the external diameter and culm wall thickness.

The objective of this study is to determine the relationship between the axial load-bearing capacity of bamboo and the non-destructive indicating variables of *Dendrocalamus sericeus* Munro bamboo. This particular species of bamboo is frequently used as a building material [34]. Additionally, the Royal Project Foundation in Thailand has marketed it as a plantation for the production of culm, with market opportunities and production potential in the area [4]. Statistical analysis was used to determine the relationship between the non-destructive test properties and axial load strength, which are statistically called independent and dependent variables, respectively. First, their relationships were obtained based on simple linear regression analysis (SLRA), which is easily applicable and has been widely used [35]. However, the analysis is limited to a single independent variable relationship, which generally provides a lower correlation (R^2^ value). Hence, multiple linear regression analysis (MLRA), which is an extension of simple linear regression analysis [36,37], was carried out to include multiple independent variables in the relationship and improve the prediction accuracy for the dependent variables. The result of this study provides a useful framework for quality control in the bamboo construction industry.

## 2. Materials and Methodology

### 2.1. Sample Preparation

**Figure 1:**
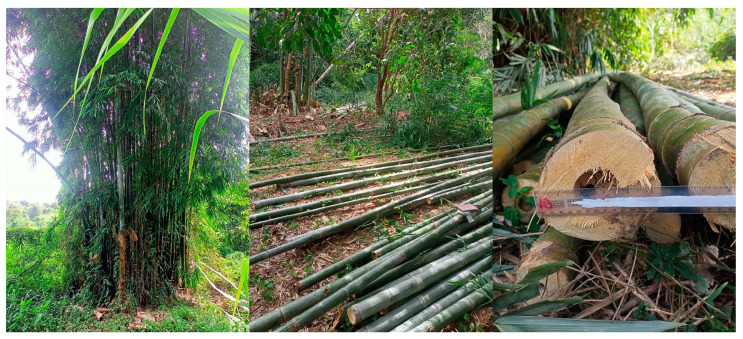
Fresh *Dendrocalamus sericeus* Munro bamboo.

**Figure 2:**
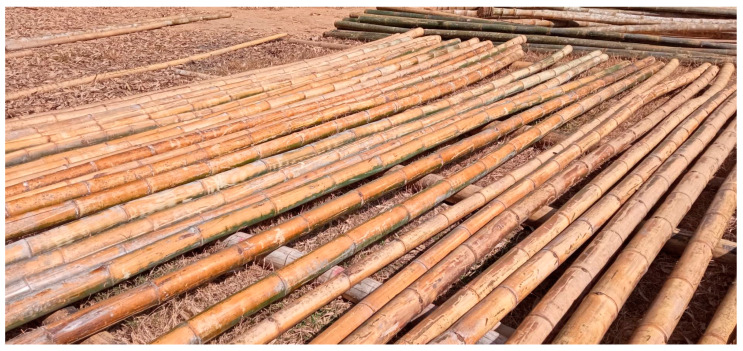
Bamboo turns from green to yellow.

**Figure 3:**
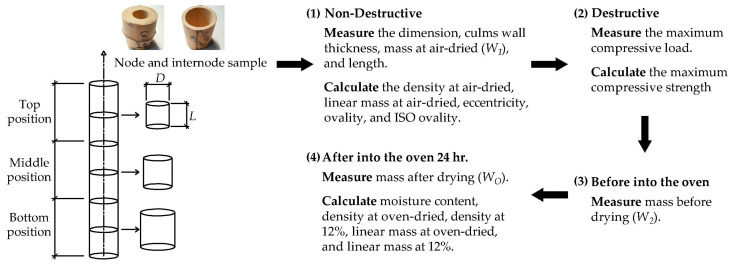
Sampling process and experiments.

The physical and mechanical properties of *Dendrocalamus sericeus* Munro, a bamboo culm from northern Thailand (Figure 1), were measured and analyzed in accordance with ISO 22157-1: 2019 [19]. All culms had a 15-day soak in a 1:1.5 mixture of boric acid and borax, a boron-based preservation treatment. The bamboo culms were next dried for a number of days in ambient air without exposure to direct sunlight in order to prevent rapid drying that could cause splitting (Figure 2), turning the bamboo color from fresh green into dry brown. One hundred and eleven pieces of *Dendrocalamus sericeus* were taken from 39 culms in order to identify samples for assessing potential indicating properties (IPs). Bamboo culm top, middle, and bottom portions were used to collect samples. wherein samples were chosen either from internode segments or node segments. The specimen lengths (*L*) were roughly equal to their exterior diameters (*D*). When all measurements and tests were performed, all specimens were air-dried. Figure 3 shows the sampling process and experiments. Due to the variation in properties along the height, the ISO 22157-1:2019 standard [19] recommends dividing the test bamboo culm into three equal parts along the length and sampling, marking “T” for (Top), “M” for (Middle), and “B” for (Bottom) for experimental evaluations.

### 2.2. Measurement and Calculation of Indicating Properties

As shown in Figure 3, first, each specimen was measured for its length (*L*), exterior diameter (*D*), wall thickness (*t*), and weight ($W_{1}$). Per specimen, the length and external diameter were measured twice, while the wall thickness was measured four times by using a digital caliper with 0.01 mm accuracy. The exterior diameter of each section was measured at the major axis (maximum diameter, *D_max_*) and its corresponding minor axis because the specimen’s section form might not be perfectly round (minimum diameter, *D_min_*). Then, an ultimate compression test was performed. The compressed specimen was then taken for the weight measurement ($W_{2}$). Next, the specimen was oven-dried for 24 h at 103 ± 2 °C, and the weight was recorded as $W_{o}$. The moisture content ($M_{c}$) at the compression test was calculated as in Equation (1).

(1) $$M_{c}=\frac{W_{2}-W_{O}}{W_{O}}\times 100\%$$

With the measured weights and dimensions, the variables that reflect “fiber densities” were next determined, including traditional density (ρ) and linear mass (q). According to their moisture content, the density and linear mass were further classified as air-dried ($\rho_{a}$ and $q_{a}$), oven-dried ($\rho_{o}$ and $q_{o}$), or at 12% moisture content ($\rho_{12}$ and $q_{12}$): a standardized moisture content according to ISO 22157-1:2019 [19]. Equations (2)–(7) illustrate how these “densities” were calculated. It should be noted that Equations (2)–(7) utilize average values for exterior diameter (*D*), length (*L*), and wall thickness (*t*).
Air-dried density:$\rho_{a}=\frac{4W_{1}}{\pi L\left(D^{2}-{\left(D-2t\right)}^{2}\right)}$(2)Oven-dried density:$\rho_{o}=\frac{\rho_{a}}{\left(1+M_{c}\right)}$(3)Density at 12% moisture content:$\rho_{12}=1.12\rho_{o}$(4)Air-dried linear mass:$q_{a}=\frac{W_{1}}{L}$(5)Oven-dried linear mass:$q_{o}=\frac{q_{a}}{\left(1+M_{c}\right)}$(6)Linear mass at 12% moisture content:$q_{12}=1.12q_{o}$(7)

The non-circularity (integrity) of the cross-section affects the compressive load capacity of the slender column [21]. The eccentricity ($e_{c}$), ovality ($O_{v}$), and ISO ovality ($d_{o}$) were used to calculate the section’s non-circularity according to ISO 19624:2018 [20]. The three “non-circularity” values were computed utilizing Equations (8)–(10). $e_{c}$ equals 0 or $O_{v}$ equals 1 only as in the case of an ideal, perfect circular cross-section [38].
Eccentricity:$e_{c}=\sqrt{1-\frac{{\left(D_{\min}\right)}^{2}}{{\left(D_{\max}\right)}^{2}}}$(8)Ovality:$O_{v}=\frac{D_{\min}}{D_{\max}}$(9)ISO Ovality:$d_{o}=\frac{\left(D_{\max}-D_{\min}\right)}{D_{avg}}=\frac{2\left(D_{\max}-D_{\min}\right)}{\left(D_{\max}+D_{\min}\right)}$(10)

### 2.3. Compressive Tests

**Figure 4:**
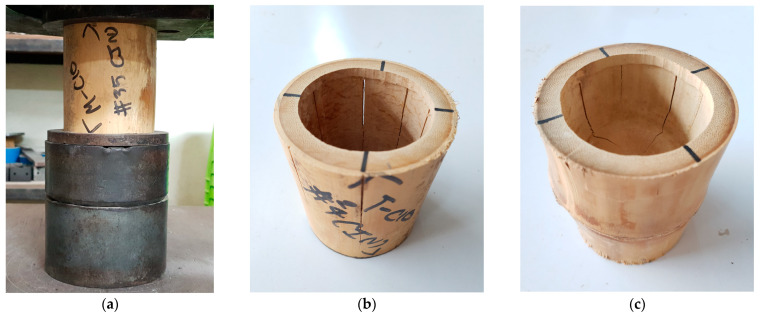
Destructive test: (**a**) compressive strength test (**b**) failure mode of internode bamboo culm, and (**c**) failure mode of node bamboo culm.

The compressive testing to failure was carried out, as shown in Figure 4, in order to establish the maximum compression load that could be used in the design. The compressive tests were conducted in accordance with ISO 22157-1:2019, clause 9 [19], with a 0.01 mm/s loading rate. The maximum compressive load ($F_{u}$) was recorded. Then, the compressive strength ($\sigma_{u}$) was calculated using Equation (11).

(11) $$\sigma_{u}=\frac{F_{u}}{A_{w}}=\frac{4F_{u}}{\pi \left(D^{2}-{\left(D-2t\right)}^{2}\right)}$$

where *A_W_* is the sectional area of the culm wall.

The failure mode for bamboo under compression, either node or internode specimens, is crack splitting. With an increase in the compressive load, splitting vertical cracks were observed when reaching the ultimate compressive load. The internode samples contained wider cracks compared to those of the node samples due to the effect of the transverse diaphragm, as shown in Figure 4.

### 2.4. Correlation Analysis between the Indicating Properties and the Compressive Strengths

The Pearson correlation coefficient (*r*) was used as a statistical measure to determine a mutual connection or dependence between the non-destructive indicating properties described in Section 2.2 and the compressive capacities ($F_{u}$ and $\sigma_{u}$) in Section 2.3. The coefficient ranges between −1 and +1, with values close to −1 or +1 indicating perfect correlation and 0 indicating no correlation. When the Pearson correlation is positive, both values are moving in the same direction, and when it is negative, the opposite is true [39,40,41]. Table 1 includes a list of the statistical correlation’s strengths.

### 2.5. Simple and Multiple Linear Regression Analysis

The compressive test results are defined as dependent variables ($y_{i}$), while the indicative qualities are defined as independent variables ($x_{i}$). According to Equation (12), simple linear regression analysis (SLRA) links the independent and dependent variables as follows:

(12) $$y_{i}=a+bx_{i}+\varepsilon_{i}$$

where a is the y-intercept term, and b is the regression coefficient representing the slope of the linear equation. ε is an error term. i is dependent variable i.

Multiple linear regression analysis involves adding more independent variables to an equation. According to Equation (13), a multiple linear regression analysis model is created [37].

(13) $$y_{i}=\beta_{0}+\beta_{1}x_{1i}+\beta_{2}x_{2i}+\ldots +\beta_{n}x_{ni}+\varepsilon_{i}$$

where $\beta_{i}$ are regression coefficients and $x_{ni}$ is independent variable n of the dependent variable i. ε is an error term. n is dependent variable n.

### 2.6. Structural Grading

Bahtiar, E.T. et al. [21,29] and Trujillo, D. et al. [43] investigated the characteristic value ($R_{k}$) and structural grading of bamboo using ISO 22156 [44]. First, the simple linear regression analysis in Equation (12) was applied. Next, the value of the 5% exclusion limit ($R_{0.05}$) was determined using Equation (14). Finally, the characteristic value was calculated using Equation (15).

(14) $$R_{0.05}={\overset{⌢}{y}}_{i}-t_{\left(\upsilon ,0.95\right)}S_{E}\;;\;S_{E}={\left(1+\frac{1}{n}+\frac{{\left(x_{i}-\bar{x}\right)}^{2}}{\sum {\left(x_{i}-\bar{x}\right)}^{2}}\right)}^{0.5}S_{r}$$

(15) $$R_{k}=R_{0.05}\left(1-\frac{2.7S_{E}}{m\sqrt{n}}\right)$$

where *m* = the response mean ($F_{u}$ or $\sigma_{u}$), *n* = the specimen size, $S_{E}$ = standard error of prediction at a given value of *x*, $S_{r}$ = standard error of regression, *x_i_* = predictor value, $\bar{x}$ = mean of predictor, and $t_{\left(\upsilon ,0.95\right)}$ = one-tail Student’s t-distribution value with υ degree of freedom for 95% probability.

## 3. Results and Discussion–Physical and Mechanical Properties

Table 2 displays the outcomes from the 111 tests. It should be mentioned that six test results were disregarded owing to a high variance.

### 3.1. Data Frequency Distribution

Skewness and Excess Kurtosis (abbreviated “Kurtosis”) are measures of the distribution’s asymmetry and tailing with respect to a normally distributed population [45,46]. Skewness and kurtosis values of zero indicate perfectly normally distributed observed data. Table 2 shows that all of the variables’ skewness ranged from −0.462 to 0.976, and kurtosis ranged from −0.821 to 0.330. Hence, the distributions of recorded data are of an acceptable normal distribution (< ±1 is perfect, < ±2 is acceptable [47]).

### 3.2. Effect of Node Inclusion and Position of the Bamboo Culm

#### 3.2.1. Dimensions

**Figure 5:**
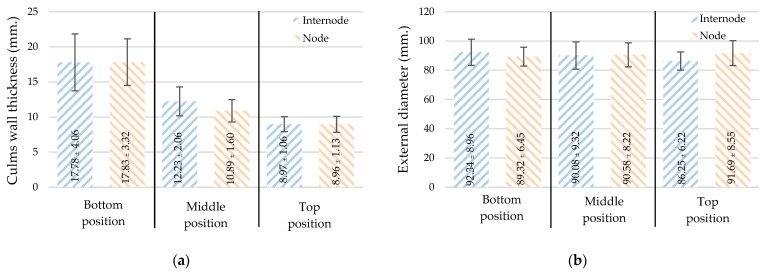
Dimensions: (**a**) culm wall thickness and (**b**) External diameter (mean ± standard deviation).

From Table 2, the exterior diameter of the test samples is between 74.60 and 109.29 mm, and the culm wall thickness is 6.78 to 27.03 mm. From Figure 5, the culm wall thickness of the bamboo measured from the bottom is greater than that measured from the top, but the external diameter is uniform along the length regardless of node and internode. The culm wall thickness of bamboo affects its ability to withstand axial loads as a result of internal fibers being compressed and compacted along a smaller cross-sectional area, with the largest cross-sectional area from the bottom gradually getting smaller at the top.

#### 3.2.2. Moisture Content ($M_{c}$)

**Figure 6:**
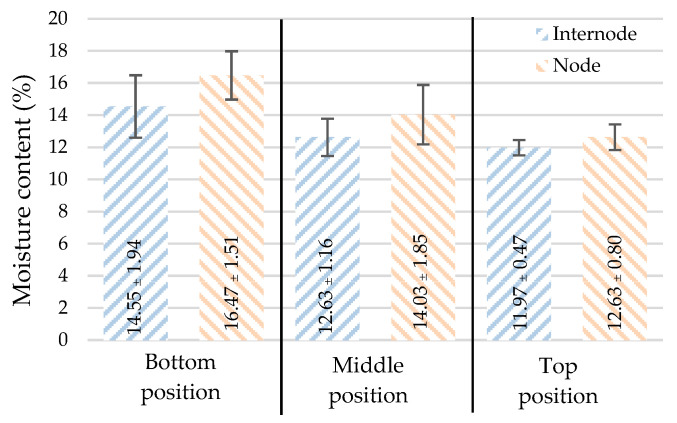
Moisture content (mean ± standard deviation).

The test results showed that the moisture content of the bamboo samples ranged from 11.07–19.70%, with an average value of 13.72% (as seen in Table 2). Figure 6 depicts the moisture content variation at various height portions. It was found that the bamboo’s bottom had the highest moisture content, which decreased as the height increased. With a node, the moisture content is greater due to the increased volume of the transverse diaphragm.

#### 3.2.3. Density (ρ) and Linear Mass (q)

**Figure 7:**
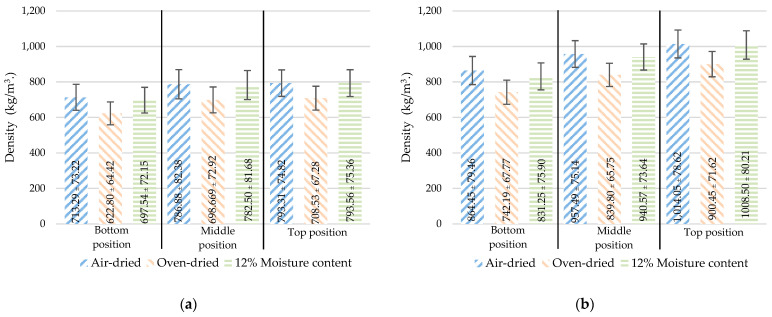
The density varied with moisture content and position: (**a**) internode, (**b**) node (mean ± standard deviation).

**Figure 8:**
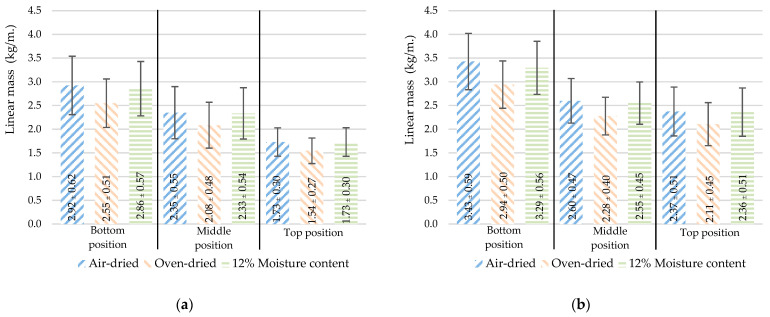
The linear mass varied with moisture contents and positions: (**a**) internode, (**b**) node (mean ± standard deviation).

Moisture absorption is a natural behavior of bamboo. As a result, the mass per volume characteristics varied depending on the amount of absorbed water. According to ISO 22157-1:2019 [19], the density (ρ) and linear mass (q) are determined based on three different moisture contents: air-dried ($\rho_{a}$, $q_{a}$), oven-dried ($\rho_{o}$, $q_{o}$), 12% moisture content ($\rho_{12}$, $q_{12}$), as respectively shown in Figure 7 and Figure 8.

At different positions, the density (Figure 7) increased with the height. However, the linear mass (Figure 8) decreased with height as the culm wall thickness of the bamboo de-creased with height, as shown in Figure 5. As the node samples have a diaphragm in the center, the overall mass of the sample increases. As a result, the node sample was greater than the internode sample in terms of both density and linear mass.

#### 3.2.4. Eccentricity ($e_{c}$), Ovality ($O_{v}$), and ISO Ovality ($d_{o}$)

**Figure 9:**
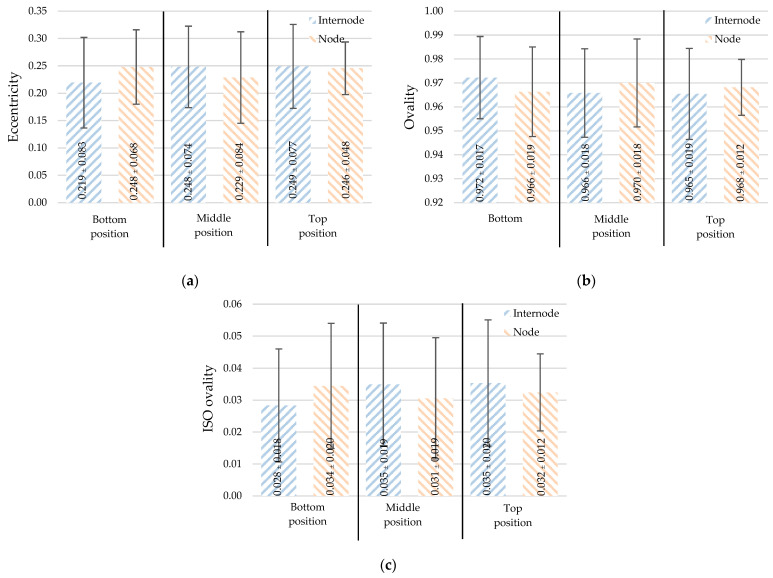
The cross-section integrity: (**a**) eccentricity, (**b**) ovality, and (**c**) ISO ovality (mean ± standard deviation).

Eccentricity and ovality are measurements of the perfect circularity of a bamboo section. From Table 2, the means and deviations for eccentricity ($e_{c}$), ovality ($O_{v}$), and ISO ovality ($d_{o}$) are 0.241 ± 0.072, 0.968 ± 0.017, and 0.033 ± 0.018, respectively. Figure 9 shows that the circularity of the section is not affected by position, node, or internode. The measurements revealed that *Dendrocalamus sericeus* Munro’s section has a high level of circularity.

#### 3.2.5. Axial Load Capacity

**Figure 10:**
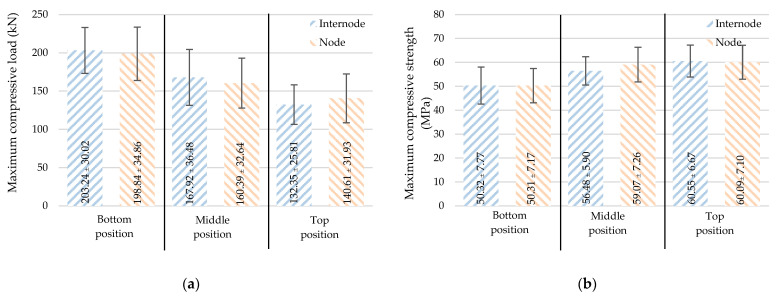
Compressive strength: (**a**) maximum compressive load and (**b**) maximum compressive strength (mean ± standard deviation).

As different parts of the bamboo culm have varying densities (or fiber contents), axial load capacity is then discussed for two values: (1) maximum compressive load ($F_{u}$) is the maximum force (kN) that the culm can sustain, and (2) maximum compressive strength ($\sigma_{u}$) is the maximum load ($F_{u}$) per loaded area (N/mm^2^ or MPa). From Table 2, the means with their respective deviation values of the capacities were 167.342 ± 41.755 kN and 55.979 ± 8.094 MPa, respectively. Figure 10 shows slight differences in the maximum compressive load and maximum compressive strength of those between the node and internode samples. Hence, the node inclusion does not affect the compressive strength of a short bamboo culm.

Figure 10a,b show the effect of position on the axial load capacity. The maximum compressive load decreases with height. The means with their corresponding deviation values at the bottom, middle, and top were 200.76 ± 32.49 kN, 164.95 ± 34.70 kN, and 135.95 ± 28.55 kN, respectively. According to Figure 10b, in contrast to the maximum compressive load, the maximum compressive strength increases with height. The figures were 60.35 ± 6.77 MPa, 57.50 ± 6.49 MPa, and 50.32 ± 7.34 MPa, respectively, for the average compressive strength at the top, middle, and bottom positions.

## 4. Correlation Analysis

### 4.1. Pearson Correlation Coefficient between Independent and Dependent Variables

Table 3 shows the one-to-one Pearson correlation coefficient (r) matrix between variables. One variable is compared mutually with only one other variable. The high value of the coefficient approaching to 1 (or −1 in the negative direction) means the two variables are very strongly or perfectly correlated, as demonstrated in Table 1. In this study, all variables were divided into two groups as independent and dependent variables. The independent variable includes all indicating properties, i.e., culm wall thickness, external diameter, moisture content, eccentricity, ovality, ISO ovality, density (air-dried, oven-dried, and 12% $M_{c}$), and linear mass (air-dried, oven-dried, and 12% $M_{c}$). The dependent variables are the maximum compressive load and maximum compressive strength.

The correlation between variables indicating parameters or non-destructive property is first investigated. From Table 3, based on the correlation levels in Table 1, the culm wall thickness has a strong correlation with moisture and linear mass (air-dried, oven-dried, and 12% $M_{c}$), moderate–substantial correlation with density (air-dried, oven-dried, and 12% $M_{c}$), and low–moderate correlation with eccentricity, ovality, and ISO ovality.

The external diameter had a substantial–very strong correlation with the linear mass (air-dried, oven-dried, and 12% $M_{c}$), a low–moderate correlation with density (air-dried, oven-dried, and 12% $M_{c}$), and a trivial correlation with moisture content, eccentricity, ovality, and ISO ovality.

The correlation of moisture content was substantial–very strong with linear mass (air-dried, oven-dried, and 12% $M_{c}$), and trivial with eccentricity, ovality, ISO ovality, and density (air-dried, oven-dried, and 12% $M_{c}$).

Subsequently, the correlation between the indicating properties (non-destructive) and grade-determining properties (destructive) is analyzed. Considering the maximum compressive load, the highest positively correlated indicating property is the linear mass, with a Pearson correlation coefficient of 0.884–0.894. It is followed in descending order by the culm wall thickness (*t*), external diameter (*D*), and moisture content ($M_{c}$). Other variables are considered to have a low–moderate or trivial correlation. The increase in the above-indicating properties will increase the maximum compressive load. For the maximum compressive strength, density is the best correlated indicating property with a Pearson correlation coefficient of 0.556–0.616. Culm wall thickness is also well correlated, but in a negative manner, with a Pearson correlation coefficient of −0.602. These findings are similar to the research by Bahtiar E. T. et al. [29]

Sectional circularity perfection measured in terms of eccentricity ($e_{c}$), ovality ($O_{v}$), and ISO ovality ($d_{o}$) is not correlated with compressive load capacities. It is due to the fact that *Dendrocalamus sericeus* Munro’s section has a high level of circularity.

### 4.2. Simple Linear Regression Analysis

**Figure 11:**
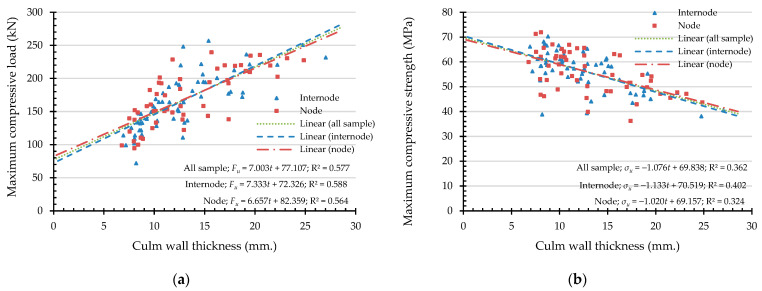
The simple linear regression analysis of bamboo’s bearing capacities with culm wall thickness as an indicating property: (**a**) maximum compressive load ($F_{u}$) and (**b**) maximum compressive strength ($\sigma_{u}$ ).

**Figure 12:**
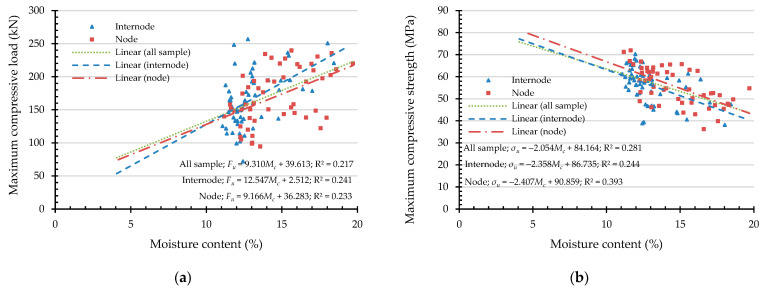
The simple linear regression analysis of bamboo bearing capacity with moisture content as an indicating property: (**a**) maximum compressive load ($F_{u}$) and (**b**) maximum compressive strength ($\sigma_{u}$ ).

**Figure 13:**
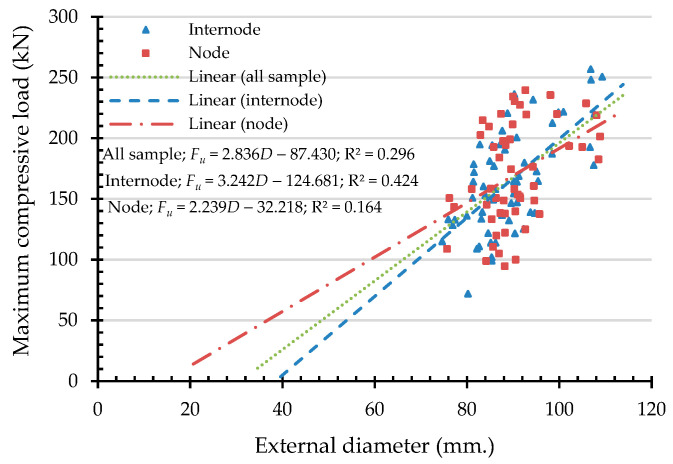
The simple linear regression analysis of bamboo maximum compressive load ($F_{u}$) with external diameter as an indicating property.

**Figure 14:**
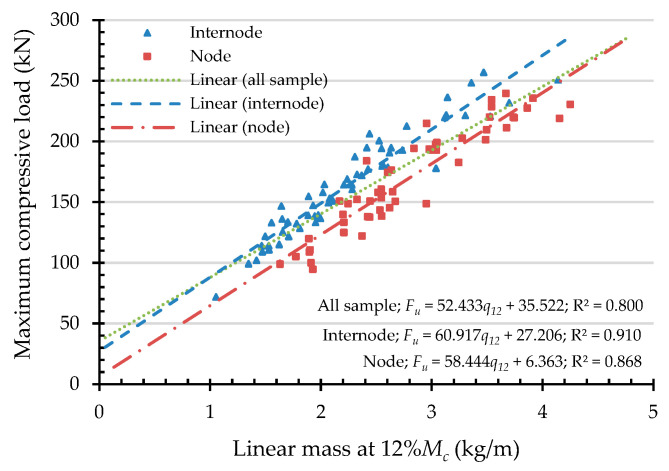
The simple linear regression analysis of bamboo maximum compressive load ($F_{u}$) with linear mass at 12% $M_{c}$ as an indicating property.

**Figure 15:**
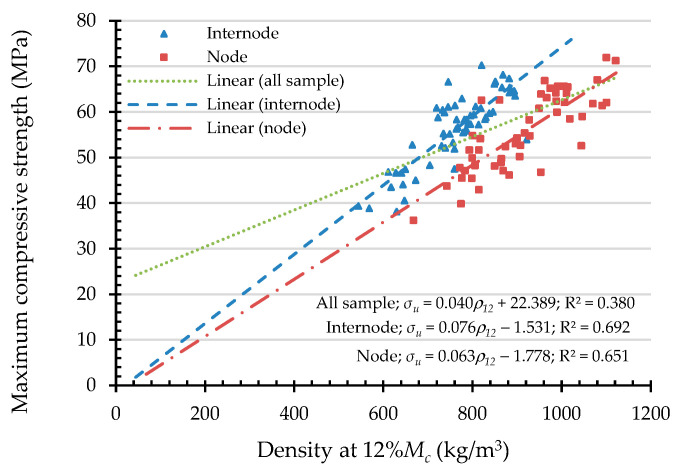
The simple linear regression analysis of bamboo maximum compressive strength ($\sigma_{u}$) with linear mass at 12% $M_{c}$ as an indicating property.

To express the compressive capacities in terms of an indicating variable, a simple linear regression (SLR) was analyzed. An independent variable was chosen from the highly correlated indicating properties according to the Pearson correlation coefficient, presented in Table 3. Hence, the independent variables of maximum compressive load are culm wall thickness, external diameter, moisture content, and linear mass at 12% $M_{c}$. In the same manner, the independent variables are culm wall thickness, moisture content, and density at 12% $M_{c}$ while the dependent variable is the maximum compressive strength. According to the simple linear regression in Equation (12), the result of the analysis of the maximum compressive load ($F_{u}$) and the maximum compressive strength ($\sigma_{u}$) are shown in Figure 11, Figure 12, Figure 13, Figure 14 and Figure 15 with the test data.

The value of R^2^ according to Figure 11, Figure 12, Figure 13, Figure 14 and Figure 15 for all samples, when the dependent variable is maximum compressive load ($F_{u}$), = 0.217–0.800, which means that the independent variable can predict the dependent variable from 21.7% to 80%. The highly predictive independent variables are linear mass at 12% $M_{c}$, culm wall thickness, external diameter, and moisture content, respectively.

For the dependent variable of maximum compressive strength ($\sigma_{u}$), the R^2^ = 0.281–0.380, which means that the independent variable can predict the dependent variable from 28.1% to 38%. The highly predictive independent variables are density at 12% $M_{c}$, culm wall thickness, and moisture content, respectively.

The analysis of the above results revealed that the independent variables used in the prediction of the maximum compressive load ($F_{u}$) had a very high R^2^ (80%). However, a rather low R^2^ of 38% was discovered for the independent factors employed in the prediction of the maximum compressive strength ($\sigma_{u}$). To improve prediction accuracy for dependent variables, the maximum compressive strength ($\sigma_{u}$) will continue to be analyzed in the multiple linear regression section.

### 4.3. Multiple Linear Regression Analysis

To increase the predictive accuracy, multiple linear regression (MLR) analysis, employing several independent variables, was adopted. However, the correlation between the independent variables employed in the analysis should be considered first, and the correlation should be low enough to prevent multicollinearity. Multicollinearity is a phenomenon that occurs when two or more predictors are correlated. If this happens, the standard error of the coefficient will increase, which will also result in some of the significant variables under study being statistically insignificant [48,49]. To check for this phenomenon, all independent variables employed in the SLR analysis were first considered for the MLR analysis. The results are shown in Table 4 as Model 1, in which the external diameter had a *p*-value > 0.05, which was not statistically significant. Then, the MLR was reanalyzed for Model 2, excluding the external diameter, which found that all independent variables had a *p*-value < 0.001 (Significance of t). As a result, Model 2 was able to predict the dependent variable’s maximum compressive load ($F_{u}$) at an R^2^ of 85.9%, which was less than that of Model 1 (R^2^ = 86.1%) but had a higher statistical accuracy. Compared with the SLR analysis, the MLR analysis for the maximum compressive load ($F_{u}$) provides a predicted value with an R^2^ that increased from 80% to 85.9%. Therefore, the MLR analysis can produce a more accurate prediction equation for the maximum compressive load ($F_{u}$).

For the MLR analysis of the maximum compressive strength ($\sigma_{u}$) (Table 5), the multicollinearity exists in the culm wall thickness variable with a *p*-value > 0.05 (NOT Sig. of t), as seen in Model 1. Then, model 2 was created without the independent variable of culm wall thickness in order to achieve statistically significant accuracy. In Model 2, all independent variables were found to have a *p*-value < 0.001 (Sig. of t), which was statistically significant. Compared with the SLR analysis with the MLR analysis for the dependent variable, the predicted maximum compressive strength (R^2^) increased from 38% to 59.6%.

### 4.4. Structural Grading of Dendrocalamus Sericeus Munro Bamboo

**Figure 16:**
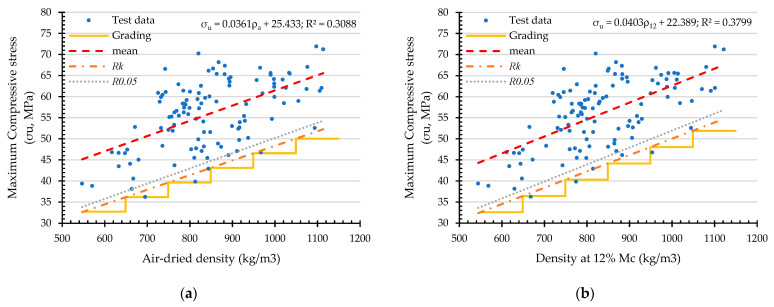
Classification of compressive strength of *Dendrocalamus sericeus* Munro based on air-dried density (**a**) and at 12% M_C_ (**b**).

**Figure 17:**
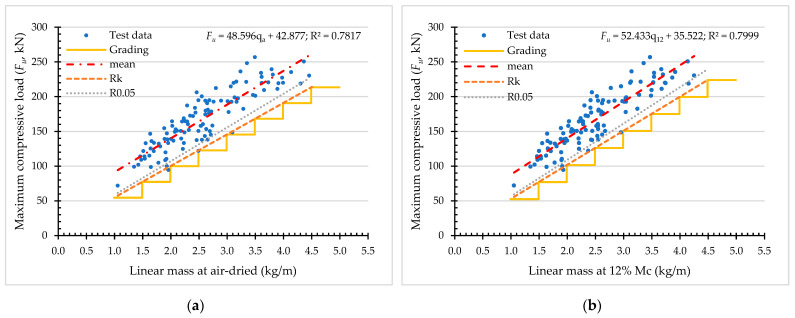
Classification of compressive load of *Dendrocalamus sericeus* Munro based on air-dried linear mass (**a**) and at 12% M_C_ (**b**).

Referring to Section 2.6, the results of structural grading for compressive strength based on density ($\rho_{a}$ and $\rho_{12}$) and linear mass ($q_{a}$ and $q_{12}$) are shown in Table 6 and Figure 16, and Table 7 and Figure 17, respectively.

As a means to assess the efficiency of each grading procedure, the ratio between the mean value and the characteristic value ($R_{k}$) is analyzed. The greater the ratio, the less efficient the structural design with higher variation, resulting in a higher margin [21]. In this study, the ratio ranges from 1.24 to 1.37 for compressive strength, and the ratio ranges from 1.19 to 1.84 for compressive load.

### 4.5. Maximum Compressive Load and Maximum Compressive Strength of Other Bamboo Species

**Figure 18:**
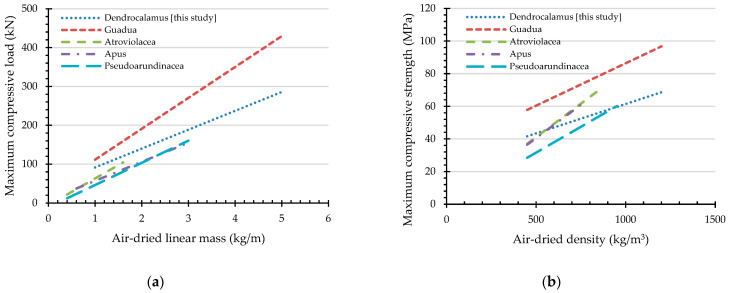
Comparisons of the compressive capacities of various bamboo species with the best indicating properties (see references in Table 8): (**a**) maximum compressive load and (**b**) maximum compressive strength.

A literature review found that regression equations have been proposed, as shown in Table 8 and Figure 18. As shown in Figure 18, increasing the air-dried linear mass and density increase the maximum compressive load and maximum compressive strength. With the same linear mass and density, *Guadua angustifolia* shows the highest capacities.

## 5. Conclusions

In this study, physical properties and compressive capacities were tested for independent and dependent variables, and statistical analysis was applied to determine their relationships. First, dependent–independent variable correlation analyses were performed using the Pearson correlation method. It was found that the relationship between culm wall thickness, external diameter, moisture content, and linear mass at 12% $M_{c}$ was strongly statistically significant with the maximum compressive load ($F_{u}$). The maximum compressive strength ($\sigma_{u}$) was well correlated to culm wall thickness, moisture content, and density at 12% $M_{c}$.

Subsequently, a simple linear regression analysis (SLRA) was applied to determine a linear relationship between an indicating property and a dependent variable. The results showed that linear mass at 12% $M_{c}$ is the best indicating property to predict the maximum compressive load ($F_{u}$) with a R^2^ of 0.800, and that density at 12% $M_{c}$ is the best indicating property to predict the maximum compressive strength ($\sigma_{u}$) with a R^2^ of 0.380. It is obviously to be seen that the two indicating properties, i.e., linear mass and density, both reflect the fiber density per length and volume, respectively. Next, multiple linear regression analyses (MLRA) were performed with several correlated independent variables to improve the prediction accuracy. Using MLRA, the R^2^ for predicting the compressive load was increased from 0.800 of the SLRA to 0.859. In addition, the R^2^ for the prediction of the maximum compressive strength ($\sigma_{u}$) was increased from 0.38 to 0.596.

From the results of this study, using some physical properties, i.e., culm wall thick-ness, external diameter, moisture content, and linear mass at 12% $M_{c}$, the compressive capacities can be estimated using simple and multi-linear regression models. The proposed equations are compared with equations from past research with other previously studied species. The equations will be useful for quality control during building construction. However, structural grading can be further processed based on reliability theory and the class of building occupation, incorporating a specific local building code.

## Figures and Tables

**Table 1:** The correlation coefficient (*r*) with the level of statistical correlation [42].

The Correlation of Coefficient (*r*)	The Level of Statistical Correlation
±0.01 to ±0.09	Trivial
±0.10 to ±0.29	Low–moderate
±0.30 to ±0.49	Moderate–substantial
±0.50 to ±0.69	Substantial–very strong
±0.70 to ±0.89	Very strong
±0.90 to ±0.99	Near perfect

**Table 2:** Descriptive statistics of dimensions and physical properties in Part 1 and results from the compressive test in Part 2.

Properties	Min	Max	Mean	SD	Skewness	Kurtosis
Part 1. Dimensions and Physical Properties						
Culm wall thickness (*t*, mm.)	6.779	27.028	12.886	4.528	0.941	0.311
External diameter (*D*, mm.)	74.603	109.290	89.821	8.013	0.739	0.330
Moisture content ($M_{c}$, %)	11.071	19.702	13.720	2.089	0.976	−0.077
Eccentricity ($e_{c}$)	0.068	0.415	0.241	0.072	−0.399	−0.065
Ovality ($O_{v}$)	0.910	0.998	0.968	0.017	−0.462	0.192
ISO ovality ($d_{o}$)	0.002	0.094	0.033	0.018	0.515	0.311
Air-dried density ($\rho_{a}$, kg/m^3^)	546.710	1113.262	847.030	124.716	0.157	−0.285
Oven-dried density ($\rho_{o}$, kg/m^3^)	485.855	1001.355	745.062	110.649	0.214	−0.212
Density at 12% Mc ($\rho_{12}$, kg/m^3^)	544.158	1121.518	834.469	123.926	0.214	−0.212
Air-dried linear mass ($q_{a}$, kg/m)	1.057	4.454	2.561	0.760	0.507	−0.382
Oven-dried linear mass ($q_{o}$, kg/m)	0.940	3.796	2.245	0.636	0.442	−0.458
Linear mass at 12% Mc ($q_{12}$, kg/m)	1.053	4.251	2.514	0.712	0.443	−0.458
Part 2. Results from the compressive test						
Maximum compressive load ($F_{u}$*,* kN)	71.982	256.934	167.342	41.755	0.132	−0.821
Maximum compressive strength ($\sigma_{u}$, MPa)	36.233	71.923	55.979	8.094	−0.358	−0.579

**Table 3:** The Pearson correlation matrix (r).

	*t*	*D*	$M_{c}$	$e_{c}$	$O_{v}$	$d_{o}$	$\rho_{a}$	$\rho_{o}$	$\rho_{12}$	$q_{a}$	$q_{o}$	$q_{12}$	$F_{u}$	$\sigma_{u}$
*t*	1	0.147	0.714 *	−0.097	0.069	−0.067	−0.36 *	−0.443 *	−0.443 *	0.792 *	0.777 *	0.777 *	0.759 *	−0.602 *
*D*		1	0.020	0.039	−0.051	0.052	0.123	0.122	0.122	0.532 *	0.559 *	0.559 *	0.544 *	−0.032
$M_{c}$			1	0.026	−0.052	0.054	0.012	−0.111	−0.111	0.691 *	0.651 *	0.651 *	0.466 *	−0.53 *
$e_{c}$				1	−0.977 *	0.974 *	0.162	0.157	0.157	0.011	0.011	0.011	−0.021	0.090
$O_{v}$					1	−1 *	−0.112	−0.104	−0.104	−0.023	−0.021	−0.021	0.002	−0.059
$d_{o}$						1	0.109	0.101	0.101	0.025	0.022	0.022	−0.001	0.056
$\rho_{a}$							1	0.992 *	0.992 *	0.165	0.174	0.174	−0.040	0.556 *
$\rho_{o}$								1	1 *	0.080	0.094	0.094	−0.096	0.616 *
$\rho_{12}$									1	0.080	0.094	0.094	−0.096	0.616 *
$q_{a}$										1	0.998 *	0.998 *	0.884 *	−0.301 *
$q_{o}$											1	1	0.894 *	−0.277 *
$q_{12}$												1	0.894 *	−0.277 *
$F_{u}$													1	−0.091
$\sigma_{u}$														1

* Correlation is significant at the 0.01 level.

**Table 4:** Multiple linear regression analysis for the dependent variable maximum compressive load ($F_{u}$ ).

Variable	Model 1				Model 2			
	Coefficients		Standardized Coefficients		Coefficients		Standardized Coefficients	
	b	Std. Error	Beta	t	b	Std. Error	Beta	t
Culm wall thickness	3.413	28.543	0.370	5.274 *	3.069	0.591	0.333	5.193 *
External diameter	0.383	0.647	0.074	1.286 ***	-	-	-	-
Moisture content	−5.834	0.298	−0.292	−5.032 *	−6.440	1.063	−0.322	−6.060 *
Linear mass at 12% Mc	44.301	1.159	0.756	8.270 *	49.567	3.463	0.845	14.315 *
Constant	57.593	5.357		2.018 **	91.530	10.889		8.405 *
	R^2^ = 0.861, Standard error of the estimate = 15.854, F = 164.254, Significance of F < 0.001				R^2^ = 0.859, Standard error of the estimate = 15.902, F = 217.130, Significance of F < 0.001			

* *p* < 0.001, ** *p* < 0.05, *** *p* > 0.05.

**Table 5:** Multiple linear regression analysis for the dependent variable maximum compressive strength ($\sigma_{u}$ ).

Variable	Model 1				Model 2			
	Coefficients		Standardized Coefficients		Coefficients		Standardized Coefficients	
	b	Std. Error	Beta	t	b	Std. Error	Beta	t
Culm wall thickness	−0.089	0.184	−0.050	−0.481 **	-	-	-	-
Moisture content	−1.683	0.360	−0.434	−4.678 *	−1.812	0.238	−0.468	−7.599 *
Density at 12% Mc	0.036	0.005	0.546	7.538 *	0.037	0.004	0.565	9.173 *
Constant	50.435	5.037		10.013 *	50.074	4.963		10.090 *
	R^2^ = 0.597, Standard error of the estimate = 5.211, F = 52.791, Significance of F < 0.001				R^2^ = 0.596, Standard error of the estimate = 5.192, F = 79.638, Significance of F < 0.001			

* *p* < 0.001, ** *p* > 0.05.

**Table 6:** Compressive strength grade classification of *Dendrocalamus sericeus* Munro based on air-dried density and at 12% M_C_.

Grade $\left(\rho_{a},\;\mathrm{kg}/m^{3}\right)$	Compressive Strength (σ, MPa)			σ$\;\mathrm{to}\;R_{k}$ Ratio	Grade $\left(\rho_{12},\;\mathrm{kg}/m^{3}\right)$	Compressive Strength (σ, MPa)			σ$\;\mathrm{to}\;R_{k}$ Ratio
	σ	$R_{0.05}$	$R_{k}$			σ	$R_{0.05}$	$R_{k}$	
550–649	47.07	35.75	34.45	1.37	550–649	46.55	35.84	34.51	1.35
650–749	50.68	39.35	37.92	1.34	650–749	50.58	39.86	38.38	1.32
750–849	54.29	42.95	41.39	1.31	750–849	54.61	43.88	42.25	1.29
850–949	57.90	46.55	44.86	1.29	850–949	58.64	47.90	46.12	1.27
950–1049	61.51	50.15	48.33	1.27	950–1049	62.67	51.92	49.99	1.25
≥1050	65.12	53.75	51.80	1.26	≥1050	66.70	55.94	53.86	1.24

**Table 7:** Compressive load grade classification of *Dendrocalamus sericeus* Munro based on air-dried linear mass and at 12% M_C_.

Grade $\left(q_{a},\;\mathrm{kg}/m\right)$	Compressive Strength (F, kN)			F$\;\mathrm{to}\;R_{k}$ Ratio	Grade $\left(q_{12},\;\mathrm{kg}/m\right)$	Compressive Strength (F, kN)			F$\;\mathrm{to}\;R_{k}$ Ratio
	F	$R_{0.05}$	$R_{k}$			F	$R_{0.05}$	$R_{k}$	
0.50–0.99	79.08	46.45	43.48	1.82	0.50–0.99	74.58	43.33	40.56	1.84
1.00–1.49	103.38	70.70	66.18	1.56	1.00–1.49	100.80	69.51	65.06	1.55
1.50–1.99	127.68	94.95	88.88	1.44	1.50–1.99	127.02	95.68	89.56	1.42
2.00–2.49	151.98	119.20	111.58	1.36	2.00–2.49	153.23	121.85	114.06	1.34
2.50–2.99	176.27	143.45	134.28	1.31	2.50–2.99	179.45	148.03	138.56	1.30
3.00–3.49	200.57	167.70	156.98	1.28	3.00–3.49	205.67	174.20	163.06	1.26
3.50–3.99	224.87	191.95	179.68	1.25	3.50–3.99	231.88	200.38	187.56	1.24
4.00–4.49	249.17	216.20	202.38	1.23	4.00–4.49	258.10	226.55	212.07	1.22
4.50–4.99	273.47	240.46	225.08	1.21	4.50–4.99	284.32	252.72	236.57	1.20
≥5.00	297.76	264.71	247.78	1.20	≥5.00	310.53	278.90	261.07	1.19

**Table 8:** Regression equations of compressive load and compressive strength of different bamboo species.

Compressive Strength	Species	Indicating Properties	Regression Equation	R^2^
Maximum compressive Load	*Dendrocalamus sericeus* (this study)	Linear mass at 12% Mc (kg/m)	$F_{u}=52.433q_{12}+35.522$	0.80
		Linear mass at air-dried (kg/m)	$F_{u}=48.596q_{a}+42.877$	0.78
		Culm wall thickness (mm)	$F_{u}=7.003t+77.107$	0.58
		External diameter (mm)	$F_{u}=2.836D-87.430$	0.30
		Moisture content (%)	$F_{u}=9.310M_{c}+39.613$	0.22
	*Guadua angustifolia* [21]	Linear mass at 12% Mc (kg/m)	$F_{u}=80.44q_{12}+25.02$	0.89
		Linear mass at air-dried (kg/m)	$F_{u}=79.57q_{a}+31.93$	0.88
		External diameter (mm)	$F_{u}=4.55D-228$	0.76
	*Gigantochloa atroviolacea* [29]	Linear mass at air-dried (kg/m)	$F_{u}=68.843q_{a}-5.525$	0.95
	*Gigantochloa apus* [29]	Linear mass at air-dried (kg/m)	$F_{u}=49.128q_{a}+7.842$	0.82
	*Gigantochloa pseudoarundinacea* [29]	Linear mass at air-dried (kg/m)	$F_{u}=57.055q_{a}+0.692$	0.95
Maximum compressive strength	*Dendrocalamus sericeus* (this study)	Density at 12% Mc (kg/m^3^)	$\sigma_{u}=0.040\rho_{12}+22.389$	0.38
		Density at air-dried (kg/m^3^)	$\sigma_{u}=0.036\rho_{a}+25.433$	0.31
		Culm wall thickness (mm)	$\sigma_{u}=-1.076t+69.838$	0.36
		Moisture content (%)	$\sigma_{u}=-2.054M_{c}+84.164$	0.28
	*Guadua angustifolia* [21]	Density at 12% Mc (kg/m^3^)	$\sigma_{u}=0.057q_{12}+30.558$	0.22
		Density at air-dried (kg/m^3^)	$\sigma_{u}=0.052q_{a}+34.432$	0.19
	*Gigantochloa atroviolacea* [29]	Density at air-dried (g/cm^3^)	$\sigma_{u}=0.08224q_{a}-0.01406$	0.74
	*Gigantochloa apus* [29]	Density at air-dried (g/cm^3^)	$\sigma_{u}=0.08124q_{a}-0.0183$	0.68
	*Gigantochloa pseudoarundinacea* [29]	Density at air-dried (g/cm^3^)	$\sigma_{u}=0.06329q_{a}-0.00448$	0.55

## Data Availability

All datasets presented in this study are included in the article.
